# Activated matriptase as a target to treat breast cancer with a drug conjugate

**DOI:** 10.18632/oncotarget.25414

**Published:** 2018-05-25

**Authors:** Gulam M. Rather, Siang-Yo Lin, Hongxia Lin, Whitney Banach-Petrosky, Kim M. Hirshfield, Chen-Yong Lin, Michael D. Johnson, Zoltan Szekely, Joseph R. Bertino

**Affiliations:** ^1^ Rutgers Cancer Institute of New Jersey, Rutgers, The State University of New Jersey, New Brunswick, NJ, USA; ^2^ Department of Medicine, Robert Wood Johnson Medical School, Rutgers, The State University of New Jersey, New Brunswick, NJ, USA; ^3^ Department of Oncology, Georgetown Medical School, Washington, DC, USA; ^4^ Department of Pharmaceutics, Ernest Mario School of Pharmacy, Rutgers, The State University of New Jersey, Piscataway, NJ USA; ^5^ Department of Pharmacology, Robert Wood Johnson Medical School, Rutgers, The State University of New Jersey, New Brunswick, NJ, USA

**Keywords:** matriptase, monomethyl auristatin E, antibody-drug conjugate, copper-free click chemistry, xenograft

## Abstract

The antitumor effects of a novel antibody drug conjugate (ADC) was tested against human solid tumor cell lines and against human triple negative breast cancer (TNBC) xenografts in immunosuppressed mice. The ADC targeting activated matriptase of tumor cells was synthesized by using the potent anti-tubulin toxin, monomethyl auristatin-E linked to the activated matriptase-specific monoclonal antibody (M69) via a lysosomal protease-cleavable dipeptide linker. This ADC was found to be cytotoxic against multiple activated matriptase-positive epithelial carcinoma cell lines *in vitro* and markedly inhibited growth of triple negative breast cancer xenografts and a primary human TNBC (PDX) *in vivo*. Overexpression of activated matriptase may be a biomarker for response to this ADC. The ADC had potent anti-tumor activity, while the unconjugated M69 antibody was ineffective in a mouse model study using MDA-MB-231 xenografts in mice. Treatment of a human TNBC (MDA-MB-231) showed potent anti-tumor effects in combination with cisplatin in mice. This ADC alone or in combination with cisplatin has the potential to improve the treatment outcomes of patients with TNBC as well as other tumors overexpressing activated matriptase.

## INTRODUCTION

The recent development of antibody drug conjugates (ADCs) that are approved by the FDA for treatment of Hodgkin disease [1], breast cancer [2] and acute myelogenous leukemia [3] has stimulated the development of other tumor target directed ADC's, and many others are being tested in the clinic with anti-tumor activity [2, 4, 5]. A key advance in this field has been the development of linkers between the antibody and toxin that allows stability in blood, but are cleaved intracellularly to release the toxin [6].

We have identified an antibody to a membrane bound and epithelial-derived protease, “activated” matriptase, in complex with its inhibitor HAI-1 as an attractive target antigen for highly selective delivery of cytotoxins to epithelial tumors and some B-cell malignancies [7–10].

Matriptase, also called MT-SP1 or epithin, is a member of the family of type II transmembrane serine proteases on the surface of the normal epithelium. It is an 80–90 kDa glycoprotein with a complex structure with regulatory mechanisms and functions [11, 12]. The enzyme contains a cytoplasmic N-terminus that mediates basolateral membrane localization in epithelial cells [13], a short transmembrane segment, and a large C-terminal region including a catalytic serine-protease domain and several non-catalytic domains (a single SEA, two CUB and four LDLRA domains). Matriptase and its endogenous inhibitor HAI-1, a type I transmembrane Kunitz type serine protease inhibitor, represent a cognate pair, commonly deregulated and ubiquitously expressed in a variety of human carcinomas in particular, breast cancer [7, 8, 14, 15]. Matriptase is synthesized as an inactive single-chain zymogen on the rough endoplasmic reticulum and travels to the plasma membrane via the Golgi apparatus [12]. The zymogen is cleaved to generate disulfide-linked-two-chain fully active enzyme through an auto-activation step; enzyme activity is regulated by binding to inhibitors (HAI-1).

Activated matriptase is expressed by various tumors of epithelial origins, including breast, prostate, ovarian, uterine, colon, cervix, stomach, pancreas, and epithelial-type mesothelioma, as well as in some B-cell lymphomas [11]. Moreover, overexpression of the enzyme is often associated with poor prognosis of multiple cancer types such as breast, prostate, endometrial, cervical, ovarian and gastric cancers [7, 16–22]. A transgenic mouse model showed that a slight increase in the matriptase-HAI-1 ratio was sufficient for the induction of spontaneous carcinoma [23]. Recent studies showed that mice with reduced levels of matriptase display a significant delay in mammary tumor formation and blunted tumor growth mediated through the HGF/MET axis [15]. Moreover, matriptase activates keratinocyte stem cell PAR-2 to elicit its pro-inflammatory and pro-tumorigenic effects. The enzyme mediates PAR-2-NF-kB inflammatory signaling and PI3K-Akt-mTor survival/proliferative signaling and may contribute to pro-tumorigenic signaling in human epithelial carcinogenesis [24]. In addition to profound roles in initiating carcinogenesis, the enzyme also plays important roles in tumor progression including invasiveness and metastasis [25–27].

The matriptase zymogen is activated by reactive oxygen species (ROS) as well as acidity [28, 29]. This is of importance, given the increase in ROS and acidic environment of solid tumors. Although most tumor antigens targeted by ADCs are discovered by gene expression profiling, such assays would fail to identify changes due to post-translational modifications, resulting in a limited identification of potential tumor antigen targets. The specific mAb (M69) used in this study was generated by purifying the activated form of the protein in complex with its inhibitor, HAI-1 [30]. The M69 antibody specifically recognizes activated matriptase and in complex with HAI-1 [31, 32].

Here, we report the synthesis and anti-tumor studies of a novel ADC targeting activated matriptase of tumor cells. The toxin is the potent anti-mitotic agent, monomethyl auristatin-E (MMAE) linked to the activated matriptase-specific monoclonal antibody (M69) via a lysosomal protease (cathepsin B) -cleavable dipeptide linker. This ADC was found to be potent and selective against multiple activated matriptase-positive epithelial carcinoma cell lines *in vitro*. Treatment of human triple negative breast cancer (TNBC) xenografts and a primary human TNBC (PDX) showed potent anti-tumor effects alone or in combination with cisplatin in mice.

## RESULTS

### Coupling of MMAE via a releasable linker to a M69 mAb to form a conjugate: huM69-MMAE

**Figure 1:**
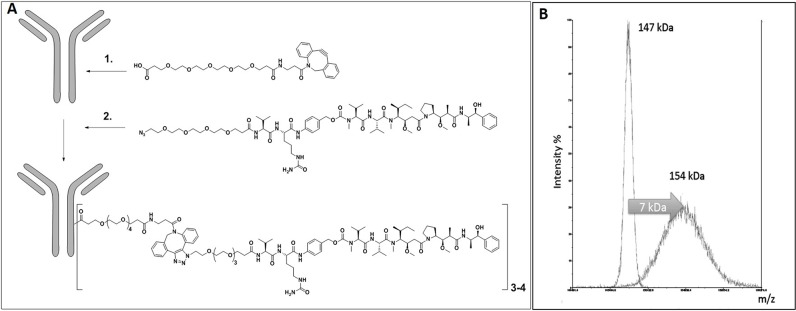
**(A)** Conjugation Technology steps. 1-Derivatization of lysine side-chains with PEG_6_-DBCO. 2-Ligand (releasable-linker MMAE) conjugation-via click chemistry. 3-4 indicates number of MMAE molecules conjugated per M69 molecule. **(B)** HR-MALDI-TOF Analysis of M69 and the conjugate formed applying the chemical steps depicted in Figure 1A.

Our releasable linker technology is based on Seattle Genetics' Valine-Citrulline-PABA linker that is cleavable by cathepsin B in the lysosomes, while showing stability in circulation [33]. We have recently improved this linker platform using copper-free click chemistry, enabling us to perform the crucial drug loading steps in a stoichiometrically controlled manner under very mild conditions. We utilized lysine side chains to conjugate the linker-drug ligand onto the surface of the antibodies instead of using temporarily reduced sulfhydryl groups of cysteine residues (Figure 1A). Since our approach does not affect disulfide bridges between cysteines, the mAb molecules remain structurally intact during the conjugation procedure, eliminating the problem with loss of activity by misfolded/dissociated mAb chains. Appropriate analytical procedures (HR-MALDI-TOF mass spectrometry) demonstrated that recent batches of ADCs meet industrial standards. We have further advanced the technology by PEGylating the drug-linker part of the conjugate on both precursors of the click chemistry that facilitates the use of very hydrophobic drug molecules. Our present toxin of choice is MMAE, an FDA approved warhead component for ADCs. The conjugation reactions were monitored by MALDI-TOF mass spectrometry showing a 7000 Da increase of the average M.W. that corresponds to an average of 3.5 drug (MMAE) molecules linked to each mAb molecule (Figure 1B).

### *In vitro* cytotoxicity of anti-matriptase antibody (M69) conjugated with monomethyl auristatin-E (MMAE)

To evaluate the *in vitro* cytotoxicity of M69-MMAE, TNBC cell lines including MDA-MB-468, MDA-MB-231 and BT549, prostate (DU145, PC3, and PC3R), and NSCL (H322 and H1299) cancers, all expressing activated matriptase were subjected to cell viability assays. These cell lines were sensitive to the ADC with IC_50_s at single digit μg/ml of the conjugate (Table 1). As an average of 3.5 molecules of MMAE are attached to each mAb (*vide supra*), the IC_50_ values are also presented as equivalent MMAE concentrations as indicated. Importantly, taxotere resistant prostate cancer (PC) cells, PC3R, were also sensitive to M69-MMAE.

**Table 1:** IC_50_ of M69-MMAE towards various types of cancer

Cancer type	Cell line	IC_50_ M69-MMAE (μg/mL)^#^	IC_50_ equivalent MMAE (pM)
Breast	MDA-MB-231	2.6 ± 0.09	52.26
	MDA-MB-468	3.4 ± 0.30	68.34
	BT549^*^	125.0 ± 10.25	837.5
Prostate	DU145	8.0 ± 3.4	160.8
	PC3	6.8 ± 0.65	136.7
	PC3R	4.0 ± 0.34	53.6
Non-small cell Lung	H322	6.3 ± 0.20	122.61
	H1299^*^	113.0 ± 5.20	757.1
Ovary	Ovcar5	16.0 ± 0.4	321.6
Pancreas	PANC1	12.0 ± 0.06	241.2
	MIA-PaCa-2	8.0 ± 0.83	160.8
Stomach	AGS	1.5 ± 0.03	30.15

^*^Matriptase-negative cell lines

^#^Mean values with standard deviation were used.

### Activated matriptase as a selective marker for response to the ADC

To determine if the presence of activated matriptase is a marker for response to the ADC, we examined four cell lines that either expressed high (MDA-MB-468 and H322) or low levels (BT549 and H1299) of activated matriptase. Table 1 shows also that the cell lines with high levels of activated matriptase are highly sensitive to inhibition by the ADC as compared to the two cell lines with low levels of activated matriptase.

### Anti-tumor activity of anti-matriptase antibody (M69) conjugated with monomethyl auristatin-E (MMAE) in two human TNBC xenografts

Prior to testing the M69-MMAE conjugate, we performed a toxicity study in nude mice (n=6), administered i.p. once or twice weekly x3, at 10mg/Kg. There was no evidence for toxicity even with the twice weekly schedule (weight loss, observation, data not shown), indicating that the construct was stable and free toxin was not released and did not target normal tissues.

**Figure 2:**
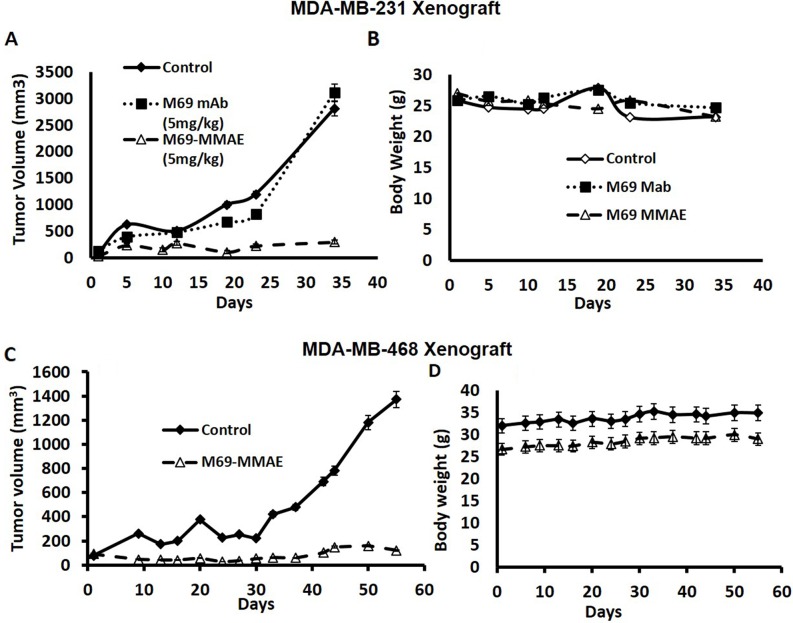
**(A-B)** Comparison of the effects of the ADC and naked M69 antibody on tumor growth of the MDA-MB-231 Xenograft using a twice weekly schedule. NSG mice were inoculated s.c. with 10 million MDA-MB-231 cells in the right flank. After tumors were palpable (100-200 mm^3^), animals were randomized into three groups (n=6) and treatments were initiated. The mice were administered the ADC (M69-MMAE) or the naked antibody (M69) i.p. twice a week X 3 weeks at 5mg/Kg. Control mice received saline. The matriptase MMAE conjugate inhibited growth of the MDA-MB-231 cell line xenograft in mice without causing weight loss or signs of toxicity. **(C-D)** Anti-tumor activity of the ADC against the MDA-MB-468 TNBC Xenograft using a twice weekly schedule. NSG mice were inoculated s.c. with 10 million tumor cells in the right flank. When tumors were palpable (100-200 mm^3^) mice were randomized into two groups (n=6) and controls were treated with saline and the experimental group with the ADC (i.p) twice a week X 2 weeks at 10 mg/Kg. The matriptase MMAE conjugate inhibited growth of the MDA-MB-468 cell line xenograft in mice without causing weight loss or signs of toxicity.

The effect of the ADC was compared to treatment with the antibody alone with a twice weekly schedule against, the MDA-MB-231 tumor, a TNBC. Figure 2A shows that the ADC had potent anti-tumor activity, while the naked antibody was ineffective. There was no evidence of toxicity (Figure 2B).

The twice weekly administration of the ADC also had potent anticancer activity against the MDA-MB-468 TNBC xenograft (Figure 2C). Again, there was no weight loss or other evidence of toxicity (Figure 2D), indicating that no significant free drug was released into the circulation from the conjugate. The caveat with regards to toxicity is that this is a mouse antibody in the mouse that recognizes only human activated matriptase, and an antibody response to matriptase would not be expected.

### Treatment of a rapidly proliferating PDX tumor with the ADC

**Figure 3:**
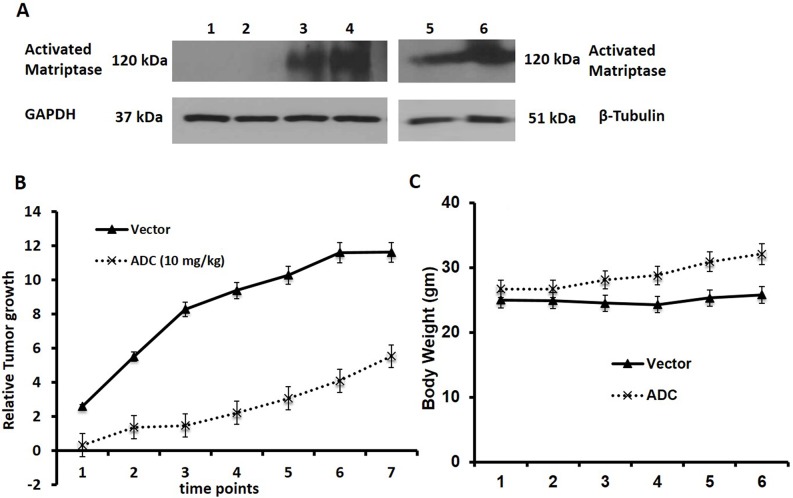
Human TNBC-PDX tumor study **(A)** Western analysis for activated matriptase expression in 6 TNBC-PDX tumors. Lane 1: 7801 LF PTMGG (SNAP); Lane 2: MGG PDX P1-T3 SNAP; Lane 3: 7853 PTP9 Mouse RT delta; and Lane 4: 7853 RT PTP9 P1; Lane 5: LF delta PT7 P0; & Lane 6: 7856PT11P1. See methods for details. **(B-C)** Anti-tumor activity of the ADC against a rapidly proliferating TNBC 7853 PDX PT9. NSG mice were inoculated s.c. with TNBC PDX in the right flank. After tumors were palpable animals were randomized into two groups (n=6) and treatments were initiated when tumors increased in size. TNBC 7853 PDX PT9 was treated with ADC (10 mg/Kg twice a week X 3 weeks). Controls were treated with saline. The M69-MMAE conjugate inhibited growth of the TNBC 7853 PDX PT9 in mice without causing weight loss or signs of toxicity.

As shown above, the presence of overexpression of activated matriptase, as detected by the M69 antibody may be a biomarker for response to the ADC. We screened 6 TNBC PDX tumors for activated matriptase expression, and found that the majority (4 of 6) expressed activated matriptase as shown by western blot (Figure 3A). We selected one of these tumors (TNBC 7853 PDX PT9, lane 4 sample) for a xenograft study to confirm the anti-tumor effects seen in the previous xenograft studies in a TNBC PDX model. The PT9 tumor originated from a patient with TNBC who had rapid progression of disease and this tumor grew rapidly in NSG mice. Treatment with the ADC twice weekly at a dose of 10 mg/Kg inhibited tumor growth over the 7 weeks of treatment (Figure 3B) again with no evidence of toxicity (Figure 3C).

### Anti-tumor activity of anti-matriptase antibody (M69) conjugated with monomethyl auristatin-E (MMAE) in combination with cisplatin in a human TNBC xenograft

**Figure 4:**
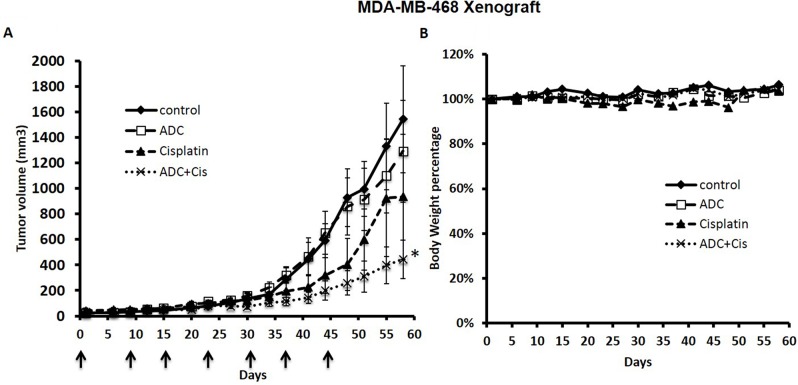
The ADC enhanced the anti-tumor response of Cisplatin against the MDA-MB-468 Xenograft NSG mice were inoculated s.c. with 4 million MDA-MB-468 cells in the right flank with an equal volume of matrigel. After tumors were palpable (100-200 mm^3^), animals were randomized into four groups (n=6) and treatments were initiated. The ADC was administered i.p. once a week at 5 mg/Kg X 6 weeks and cisplatin at 2 mg/Kg once a week X 6 weeks. Control mice received saline i.p. once a week. The matriptase-MMAE conjugate inhibited growth of the MDA-MB-468 cell line xenograft in combination with cisplatin in mice **(A)** without causing weight loss or signs of toxicity **(B)**. ^*^ P<0.02 compared with control.

We tested a weekly dose of the ADC alone and in combination with cisplatin to treat the TNBC MDA-MB-468 xenograft in NSG mice. Although with this weekly dose schedule the ADC alone had a minimal anti-tumor effect, it markedly enhanced the anti-tumor effect of cisplatin without toxicity (Figure 4).

## DISCUSSION

Studies of matriptase knockout mice have shown that matriptase plays an important role in epidermal barrier function, terminal epidermal differentiation hair follicle development, and thymic homeostatis [34]. Lack of matriptase in mutant mice is lethal postnatally due to dehydration resulting from deficiency in skin barrier function. The proteolytic cascade of the enzyme mediates the processing of profilaggrin that is crucial for forming the intact epidermal barrier [34, 35]. The protease activity of matriptase was found to be important in the formation and integrity of the intestinal epithelial barrier as shown by the studies of hypomorphic mice [36]. In addition, mutations in the human matriptase gene, ST14, are associated with an autosomal recessive skin ichthyosis characterized by compromised barrier function and hair follicle abnormality [37].

Targeting matriptase in tumor cells expressing high levels of activated matriptase with a monoclonal antibody that recognizes the activated matriptase-HAI-1 complex is a selective target for antibody delivered toxins. Low levels of matriptase are expressed in the skin layer, hair follicle cells, and monocytes [10]. Thus, even if the ADC affected these tissues, short term ADC administration should not lead to major toxicity, and in the case of monocytes may even enhance anti-tumor activity, as macrophages derived from monocytes can excrete cytokines that stimulate tumor growth [38].

In this paper, we show that the M69 antibody coupled to MMAE potently kills TNBC cells expressing activated matriptase, both *in vitro* and *in vivo*. The M69 antibody alone, had no antitumor activity (Figure 2A). Most tumors express high levels of activated matriptase [10, 15] and levels were shown to increase in gastric cancer as a function of stage [9]. Therefore, it is likely that the ADC would be most effective not only in TNBC but also in other advanced stage epithelial tumors that are usually not curable with current treatments. Further, expression of activated matriptase, which occurs in a high percent of epithelial cancers, would be a marker for response. As cisplatin or carboplatin are used to treat TNBC with some success, the enhancement of the activity of cisplatins by the ADC, as shown, could lead to major improvement of outcomes in these patients. As noted, the M69 antibody is a mouse antibody specific for human matriptase, therefore we have generated a chimeric mouse human antibody for future studies, including toxicity in primates.

Matriptase plays important roles in both initiating tumor developments by triggering HGF/MET and PAR-2 or PI3K-Akt-mTor mediated signaling as well as tumor progression, including invasiveness and metastasis [15, 24, 39]. Targeting activated matriptase-positive tumor cells should eliminate those cells that have the propensity to initiate tumor progression driven by HGF/MET and PAR-2 mediated pathways as well as those with potential to acquire invasive and metastatic phenotypes that are driven by an acidic microenvironment.

The acidic environment of tumor sites is mainly caused by overproduction of lactic acid and increases in H^+^ levels by proton pump activity [40–44]. Acidic tumor microenvironments play crucial roles in tumor progression, development of treatment resistance, and escape from immune surveillance [45, 46]. Thus, enhanced matriptase activation induced by acidified tumor sites, may be specifically targeted by the M69-MMAE antibody conjugate.

The ability of M69-MMAE to selectively target tumor cells with expression of activated matriptase, alone or in combination with cisplatin, has the potential to improve the treatment outcomes of patients with TNBC as well as other tumors overexpressing activated matriptase.

## MATERIALS AND METHODS

### Animals

Experiments were conducted in accordance with the Rutgers Cancer Institute of New Jersey Animal Care and Use Committee guidelines (protocol number 15-040). NOD/SCID/IL2 receptor gamma chain null (NOD/SCID/IL2gnull, NSG) mice were obtained from the Jackson Laboratory (Bar Harbor, ME).

### Materials

For cell culture, RPMI 1640 media, Dulbecco's modified Eagle's medium (DMEM) and fetal bovine serum were from Invitrogen (Fisher Scientific).

### Anti-matriptase antibody (M69)

The M69 mAb against the activated matriptase-HAI-1 complex was generated as reported [30].

### Cell culture

Breast cancer cells (MDA-MB-468 and BT549), Prostate cancer cells (DU145, PC3 and PC3R) and non-small cell lung cancer (H322 and H1299) cells were cultured in RPMI 1640 media. Breast cancer cells (MDA-MB-231), Ovarian cancer cells (Ovcar5), Pancreatic cancer cells (PANC1 and MIA-PaCa-2), and gastric cancer cells (AGS) were cultured in DMEM; both media contained 10% fetal bovine serum. All the cell lines were obtained from American Type Culture Collection (ATCC) and were checked for mycoplasma by MycoAlert™ mycoplasma detection kit (Lonza USA) before starting any experiment.

### Western blotting

Cells were cultured and scraped into a micro centrifuge tube. After brief centrifugation, cell pellets were lysed in 20 mM Tris and 1% Triton X-100, pH7.4, containing a commercial protease inhibitor cocktail (Roche) and 1 mM DTNB (5,5′-Dithio-bis (2-nitrobenzoic acid). Due to the interference of the DTNB in protein estimation, proteins were loaded as per the equal volume and resolved by 10% SDS-PAGE and transferred onto a nitrocellulose membrane (Bio-Rad Laboratories). Lysates were immediately diluted in 5X sample buffer (250 mM Tris-HCl, pH6.8, 30% glycerol, 10% SDS, 0.02% bromophenol blue). The sample buffer does not contain a reducing agent, and samples were not boiled prior to SDS-PAGE, since reducing agents destroy the epitopes recognized by the mAbs, and boiling disrupts the matriptase/HAI-1 complexes. After blocking the membrane with 5% non-fat dry milk prepared in Tris buffered saline + 0.1% tween-20, the membrane was incubated with the desired primary antibody according to manufacturer's directions at 4°C overnight. The membrane was washed thrice in Tris buffered saline containing 0.1% tween-20 and incubated for two hours at room temperature with the appropriate peroxidase-conjugated secondary antibody. Bands were visualized using an enhanced chemiluminescence kit (Pierce). Anti-glyceraldehyde 3-phosphate dehydrogenase (GAPDH) was purchased from Millipore. Anti-beta-tubulin and anti-mouse secondary antibody were purchased from Santa Cruz Biotechnologies.

### Cytotoxicity assay

Seven thousand cells per well were plated in RPMI 1640/DMEM media (Gibco) supplemented with 10% FBS (Invitrogen). After overnight culture, spent media was removed and fresh media containing drug was added and plates were incubated for different time periods. To assess cell viability of the cancer cell lines at the end of the experiment, the 3-(4,5-dimethylthiazol-2-yl)-5-(3-carboxymethoxyphenyl)-2-(4-sulfophenyl)-2H- tetrazolium inner salt (MTS) assay was performed according to manufacturer's protocol with the Cell Titer 96 Aqueous One Solution protocol (Promega, Madison, WI). The cytotoxicity data were further analyzed using GraphPad Prism 4 software (GraphPad Software Inc., CA). The 50% inhibitory concentration (IC_50_; the drug concentration required to obtain 50% cell kill compared to control) was determined using the non-linear regression curve fit of the graphs drawn by GraphPad Prism 4 software. All experiments were performed in triplicate wells, and all experiments were repeated at least three times.

### Animal studies

Anti-tumor activity of ADC for the xenograft studies was evaluated using MDA-MB-468 and MDA-MB-231 cells. Cells (4-10×10^6^) in 100 μL of PBS were injected subcutaneously into the right flank of 6-week-old NOD/SCIDγ (NSG) female mice. Once tumors were palpable, the mice were randomized to different groups. Mice were treated i.p. with anti-matriptase antibody conjugated with MMAE or the ADC in combination with cisplatin. Controls used were M69 (anti-matriptase) antibody and saline. Tumor size and body weights were measured twice a week and the tumor volume was calculated using the formula (length × width^2^)/2. Results are presented as mean ± SEM.

### Antitumor activity of the ADC in a human TNBC PDX model

NSG mice were inoculated s.c. with the TNBC PDX (PT9) in the right flank. After tumors increased in size, animals were randomized into two groups (n=6) and treatments were initiated. Mice were treated i.p. with anti-matriptase antibody conjugated with MMAE (ADC). Controls used saline. Tumor size and body weights were measured twice a week (represented as time points) and tumor volumes were calculated using the formula (length x width^2^)/2. Results are presented as mean ± SEM.

### Statistical analysis

All *in vitro* experiments were performed three times, and each experiment was done in triplicate. Statistical analysis was performed using GraphPad Prism software. In all cases, ANOVA followed by two-tailed, unpaired Student *t* tests were performed to analyze statistical differences between groups. *P* values of <0.05 were considered statistically significant.
